# Aluminum Chloride-Induced Planarian Model with Cognitive Impairments Through Neuronal Damage, Neurotransmitter Imbalance, Apoptosis, and Oxidative Stress

**DOI:** 10.3390/ijms27209364

**Published:** 2026-10-21

**Authors:** Qingxian Wang, Shixue Wang, Zhenbiao Xu, Linxia Song, Kexin Yang, Chunmei Zhang, Xiaorui Cheng

**Affiliations:** 1 Innovative Institute of Chinese Medicine and Pharmacy, Shandong University of Traditional Chinese Medicine, Jinan 250355, China; 2 School of Life and Medicine, Shandong University of Technology, Zibo 255000, China

**Keywords:** Alzheimer’s disease, planarian, Aluminum chloride, cognitive impairment, high-throughput screening

## Abstract

The prevalence of Alzheimer’s disease has significantly increased, which will place a heavy burden on families and society. Cognitive impairment is a typical feature of Alzheimer’s disease. Rodent models play a key role in AD research, but their considerable expense and slow development pose challenges to the pace of therapeutic discovery. Here, we established a new model of cognitive dysfunction in planarians using AlCl_3_. After exposure to 50 μM AlCl_3_ for 48 h, the planarians exhibited cognitive dysfunction in the phototaxis test, dual-stimulus decision test and T-maze test. Immunohistochemical and immunofluorescence experiments confirmed that AlCl_3_ damaged the neural structure of planarians. Moreover, AlCl_3_ disrupted the balance of neurotransmitters, leading to cell apoptosis and oxidative stress. Finally, we confirmed that donepezil alleviated the cognitive impairment caused by AlCl_3_. This indicates that the model we established for cognitive dysfunction in planarians was feasible. However, planarians lack the amyloid-β plaques and tau neurofibrillary tangles typical of AD. Therefore, the planarian model can be considered a promising complementary platform for high-throughput screening of candidate drugs for cognitive impairment.

## 1. Introduction

Alzheimer’s disease (AD) is a neurodegenerative disease, and cognitive decline is its typical characteristic [1]. In addition to the typical amyloid-β (Aβ) deposition in senile plaques and hyperphosphorylated tau protein in neurofibrillary tangles, oxidative stress and neuroinflammation are also involved in the pathogenesis of AD [2,3]. The three major factors contributing to AD are age, genetics and family history. The greatest risk factor for AD is age [4]. The world has entered an aging society, and the prevalence of AD has significantly increased. The number of patients with AD is expected to reach 130 million worldwide by 2050, which will place a heavy burden on families and society as a whole [5]. Due to the complex and incompletely understood etiology of AD, currently approved drugs such as donepezil can only ameliorate some AD symptoms but cannot prevent its progression [6,7]. Lecanemab and Aducanumab, approved by the FDA, target Aβ deposits and have shown certain therapeutic effects in the early stage of AD [8,9]. However, the treatment cost is relatively high [10]. Hence, the treatment of AD remains an unsolved problem. Rodent models play a key role in AD research, providing deep mechanistic insights. However, their considerable expense and slow development pose challenges to the pace of therapeutic discovery [11,12,13,14]. Other animal models, such as zebrafish models, Drosophila melanogaster models and Caenorhabditis elegans models, have mitigated this problem to a certain degree [15]. However, there is a clear need for alternative AD models that are cost-effective, support high-throughput screening, and can quickly identify potent compounds with therapeutic promise.

Planarians are free-living platyhelminths. They have a centralized brain with true synaptic transmission and share the majority of neurotransmitters that occur in vertebrate brains [16,17,18]. Planarians occupy an intermediate position between zebrafish and nematodes [19]. Planarians exhibit remarkable regenerative abilities, making them an ideal model for studying regeneration [20,21,22,23]. As aquatic flatworms, planarians can respond rapidly to the external environment. They have become a model organism for the detection of toxic substances and neurotoxicology research [19,24,25,26]. Furthermore, it has been reported that planarians possess cognitive functions [27,28,29]. Planarians display a number of behaviors, such as phototaxis, thermotaxis and decision-making, in response to external stimuli. Planarians can adjust behavioral features in response to stimulus conditions by integrating multiple external signals in the brain [28]. Furthermore, planarians are inexpensive, reproduce rapidly and are suitable for high-throughput screening. These features make them a useful complementary system for preliminary screening of compounds targeting cognitive impairment.

Aluminum (Al) is widely used in both industry and daily life. It can easily enter the human body via water and food. When Al enters the body, it can cause a series of adverse effects, especially on the nervous system [30,31]. However, there is controversy over whether aluminum directly causes AD [32]. Nevertheless, AlCl_3_ has been used as an experimental inducer of cognitive impairment and AD-related neuropathological changes in rats and zebrafish, and it has been widely applied in AD research [33,34,35,36].

In this work, in order to overcome some shortcomings of rodent AD models, we attempted to establish a new planarian model with cognitive impairment induced by AlCl_3_. We used a phototaxis test, a dual-stimulus decision test and a T-maze test to examine the locomotor capacity, response ability and cognitive functions of planarians. The damage to the neurons of planarians was observed through immunofluorescence and immunohistochemistry. Meanwhile, we found that after exposure to AlCl_3_, the planarians exhibited neurotransmitter imbalance, cell apoptosis and oxidative stress. Finally, we used donepezil to treat the planarian model induced by AlCl_3_. The results showed that donepezil could significantly alleviate the cognitive impairment in planarians caused by AlCl_3_. This indicates that the model we established for cognitive dysfunction in planarians was feasible.

However, as an invertebrate model, planarians lack the Aβ plaques and tau neurofibrillary tangles typical of AD, and their nervous system is simpler than that of mammals. Therefore, the planarian model can be considered a promising complementary platform for high-throughput screening of candidate drugs for cognitive impairment, rather than a full replacement for mammalian AD models or clinical studies. Findings obtained in this model require further validation in mammalian models and clinical research.

## 2. Results

### 2.1. AlCl_3_ Damages the Survival of Planarians

**Figure 1:**
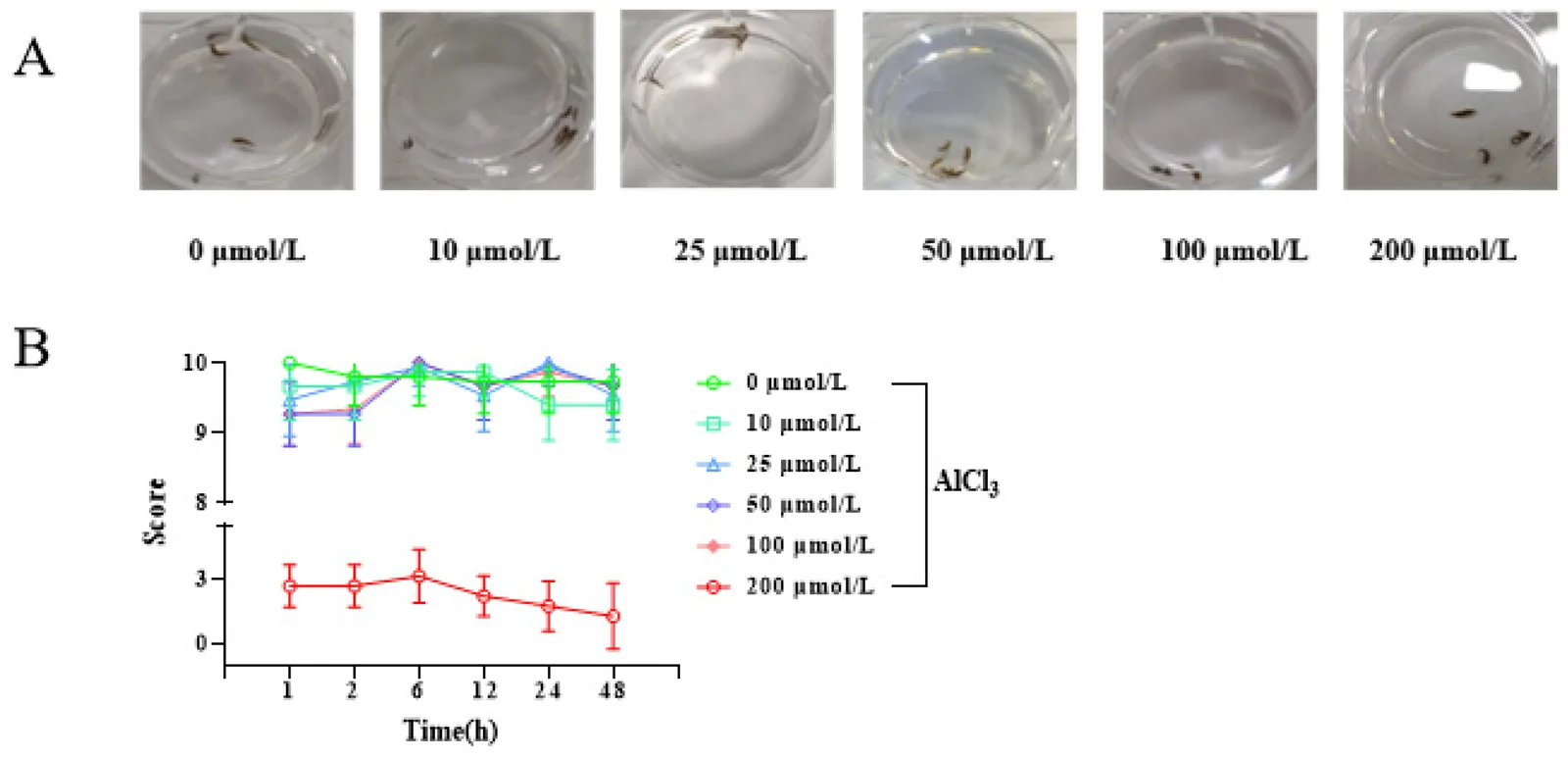
The influence of AlCl_3_ on the survival status of planarians. (**A**) Digital photos of planarians in AlCl_3_ solution at 48 h. (**B**) Score of planarian status. Mean ± S.D., N = 3, *n* = 5, GraphPad Prism 8.4.3.

We evaluated the influence of AlCl_3_ on the survival status of planarians using the scoring criteria for planarian status. The scoring criteria were established based on the literature [37,38] (Table 1). As the concentration of AlCl_3_ increased from 0 to 200 μM over 48 h, the planarians exhibited more severe toxicity (Figure 1A). The survival status scores of the planarians revealed that at concentrations below 100 μM, AlCl_3_ had no significant effect on the state of the planarians within 48 h. However, at a concentration of 200 μM, the scores of the planarians decreased significantly, and the planarians exhibited a distinct C-shaped form but did not die within 48 h (Figure 1B). Higher concentrations of 400, 800, and 1000 μM were also used, and the toxicity was further enhanced (see Supplementary File Figure S1). This indicates that at concentrations above 100 μM, the toxic effect of AlCl_3_ on planarians was too severe for these concentrations to be used in studies of nerve function impairment in planarians.

### 2.2. AlCl_3_ Impairs the Cognitive Function of Planarians

In order to investigate the impact of AlCl_3_ on the cognitive function of planarians, we used the XR-Fish2 software to record the movement trajectories of the planarians. We conducted the T-maze test, phototaxis test and dual-stimulus decision test to evaluate the influence of AlCl_3_ on planarians’ cognitive function.

**Figure 2:**
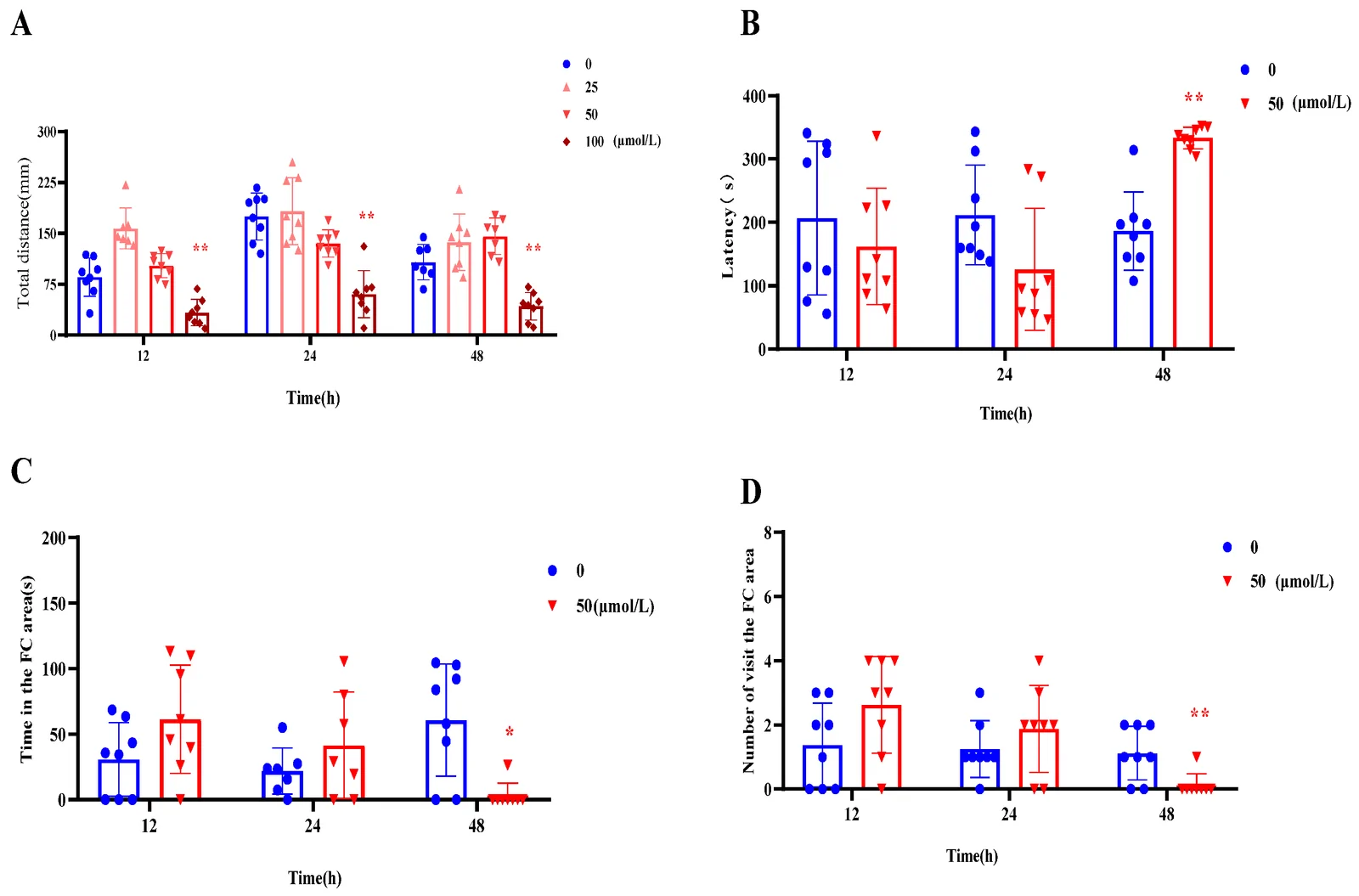
AlCl_3_ exposure induces learning and memory impairments in planarians. (**A**) The total distance travelled by planarians at different AlCl_3_ exposure times. (**B**) The latency to enter the FC area. (**C**) Duration of stay in the FC area. (**D**) Number of visits to the FC area. Mean ± SD, *n* = 8. * *p* < 0.05 and ** *p* < 0.01 vs. the 0 μmol/L AlCl_3_ group, compared among multiple groups using one-way ANOVA followed by Dunnett’s multiple comparisons test, and compared between two groups using Student’s *t*-test. GraphPad 8.4.3.

Without affecting the survival status of planarians, we investigated the influence of AlCl_3_ on the cognitive functions of planarians. We set up four groups within the range of 0 to 100 μM. We then conducted the T-maze test. The movement trajectory diagram of planarians is shown in Figure S2. After exposure to AlCl_3_ for 12, 24, and 48 h, the total distance travelled by planarians exposed to 100 μM significantly decreased (Figure 2A, *p* < 0.01), while there were no significant changes in the other concentration groups. Thus, we chose the highest concentration of 50 μM, which had no effect on movement ability, for the subsequent test. The latency for planarians to enter the feeding area (FC) showed no significant difference at 12 h and 24 h (Figure 2B). However, at 48 h, the latency was significantly prolonged (Figure 2B, *p* < 0.01). The time spent in the FC area and the number of entries into the FC area were significantly shortened and decreased at 48 h, but there was no difference at 12 and 24 h (Figure 2C, *p* < 0.05; Figure 2D, *p* < 0.01). These results indicate that 50 μM AlCl_3_ exposure for 48 h impairs the learning and memory abilities of planarians.

**Figure 3:**
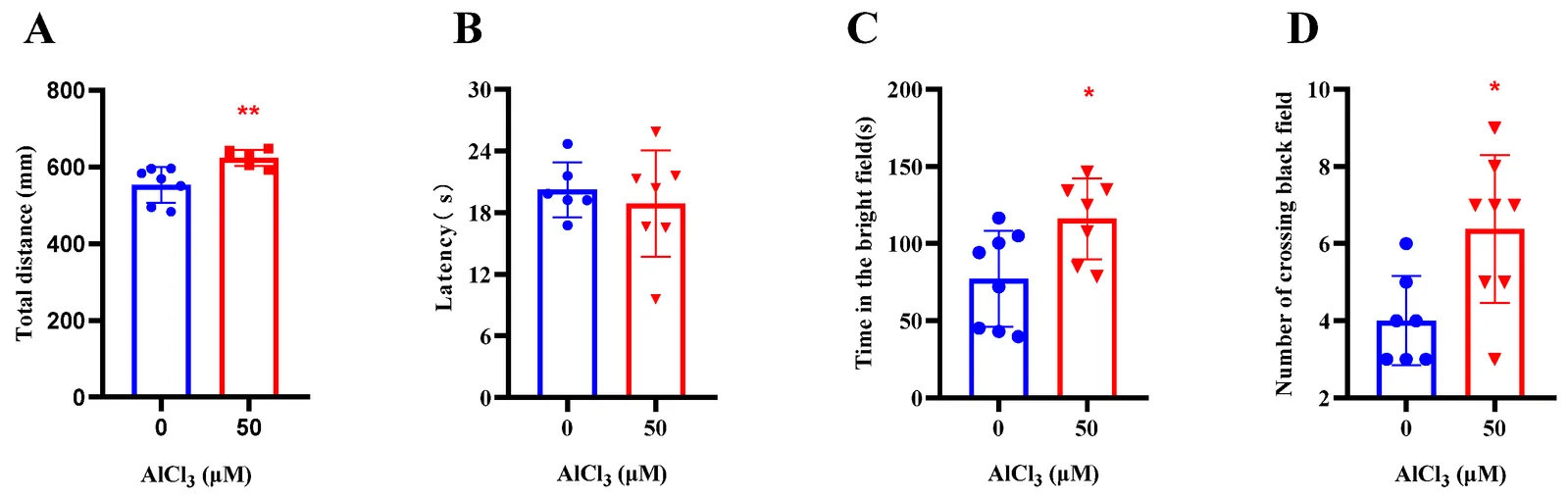
AlCl_3_ exposure induces passive avoidance impairment in planarians. (**A**) The total distance travelled by planarians. (**B**) Latency to enter the dark chamber. (**C**) Duration of stay in the bright field. (**D**) Number of crossings into the dark chamber. Mean ± SD, *n* = 8. * *p* < 0.05 and ** *p* < 0.01 vs. the 0 μmol/L AlCl_3_ group, Student’s *t*-test, GraphPad 8.4.3.

We used the phototaxis test to assess the passive avoidance ability of planarians exposed to 50 μM AlCl_3_. The movement trajectory diagram of the planarians is shown in Figure S3. Planarians have a natural tendency to avoid strong light and exhibit negative phototaxis. The total distance travelled by planarians after exposure to 50 μM AlCl_3_ for 48 h was significantly increased (Figure 3A, *p* < 0.01). Additionally, there was no difference in the latency period to enter the dark chamber (Figure 3B). The time spent in the bright field was significantly prolonged (Figure 3C, *p* < 0.05). The number of crossings into the black field was significantly increased (Figure 3D, *p* < 0.05). These results indicate that 50 μM AlCl_3_ exposure for 48 h impaired the passive response ability of planarians.

**Figure 4:**
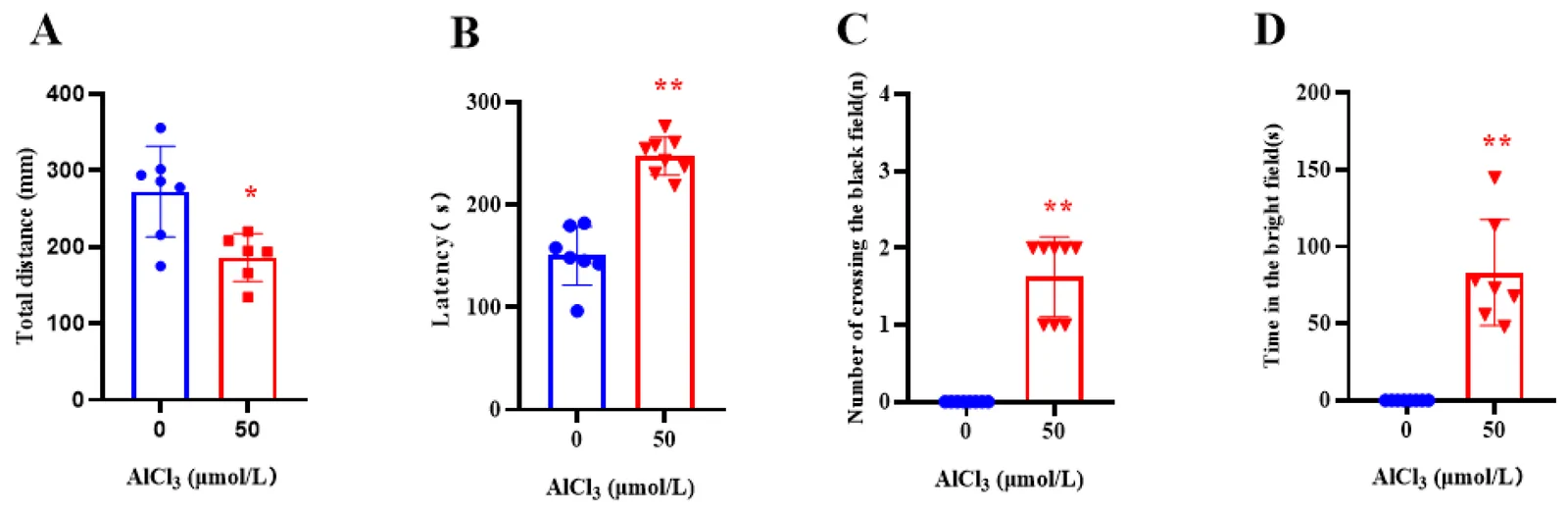
AlCl_3_ exposure impairs decision-making behavior in planarians. (**A**) The total distance travelled by planarians. (**B**) Latency of decision-making. (**C**) Duration of stay in the light chamber. (**D**) Number of crossings into the dark chamber. Mean ± SD, *n* = 8. * *p* < 0.05 and ** *p* < 0.01 vs. the 0 μmol/L AlCl_3_ group, Student’s *t*-test, GraphPad 8.4.3.

Planarians can integrate multiple external stimuli to make decisions. The dual-stimulus decision test was used to evaluate the impact of AlCl_3_ on planarians. The movement trajectory diagram of the planarians is shown in Figure S4. Light is a stimulus that planarians dislike, and food is the stimulus that planarians prefer. We observed the responses of planarians to the two different stimuli. After exposure to 50 μM AlCl_3_ for 48 h, the total distance travelled by planarians was significantly decreased (Figure 4A, *p* < 0.05). The latency time to enter the dark field was significantly prolonged (Figure 4B, *p* < 0.01). The time spent in the light field and the number of crossings into the dark chamber were significantly increased (Figure 4C, *p* < 0.01; Figure 4D, *p* < 0.01). The results indicate that exposing planarians to 50 μM AlCl_3_ for 48 h can impair their decision-making behavior.

### 2.3. AlCl_3_ Damages the Nervous System of Planarians

**Figure 5:**
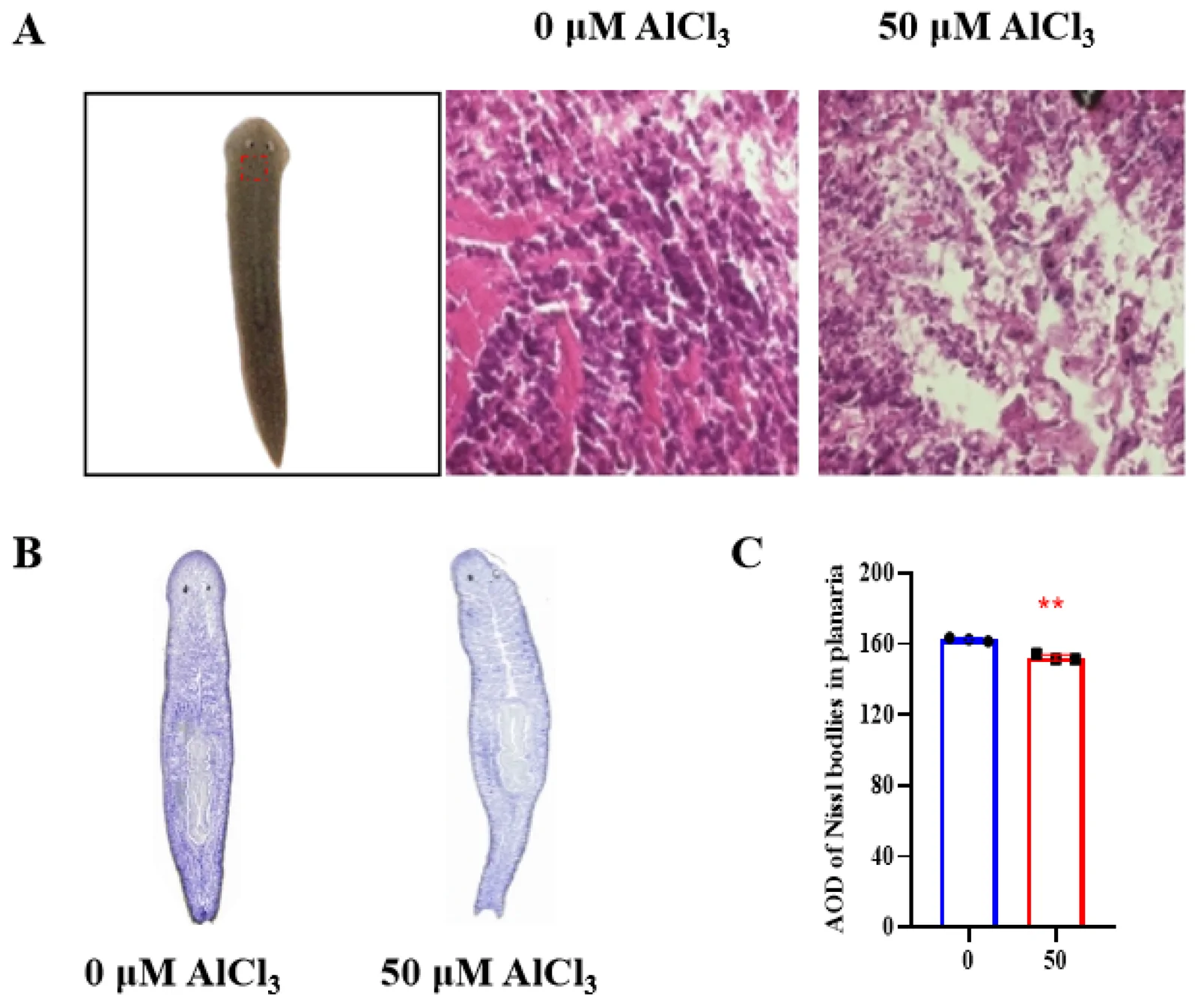
AlCl_3_ triggered an inflammatory response and damaged the neurons of planarians. (**A**) Hematoxylin and eosin (HE) staining (40×); (**B**) Nissl staining; and (**C**) average optical density (AOD) of Nissl bodies in planarians. Data are presented as the mean ± SD. ** *p* < 0.01 vs. the 0 μmol/L AlCl_3_ group, Student’s *t*-test, GraphPad 8.4.3.

We selected tissue from the middle of the planarian’s head for HE staining. Under normal conditions, cells in the planarian tissues were normal in size and morphology. The cell nuclei were large and round, and their structures were clearly visible. After exposure to 50 μM AlCl_3_ for 48 h, the cell arrangement was disordered and exhibited varying degrees of pyknosis (Figure 5A). This indicates that 50 μM AlCl_3_ caused inflammation in the planarians.

Nissl staining is a technique for staining nervous tissue. When neurons were damaged, the number of Nissl bodies decreased, and Nissl bodies even disappeared. Nissl staining showed that the Nissl bodies were large in volume and purple-blue in colour under healthy conditions. However, after exposure to 50 μM AlCl_3_ for 48 h, the purple-blue colour became lighter and the number of Nissl bodies decreased (Figure 5B). The average optical density (AOD) of Nissl bodies in planarians exposed to 50 μM AlCl_3_ for 48 h was significantly decreased (Figure 5C, *p* < 0.01). This result demonstrates that 50 μM AlCl_3_ damages neurons.

**Figure 6:**
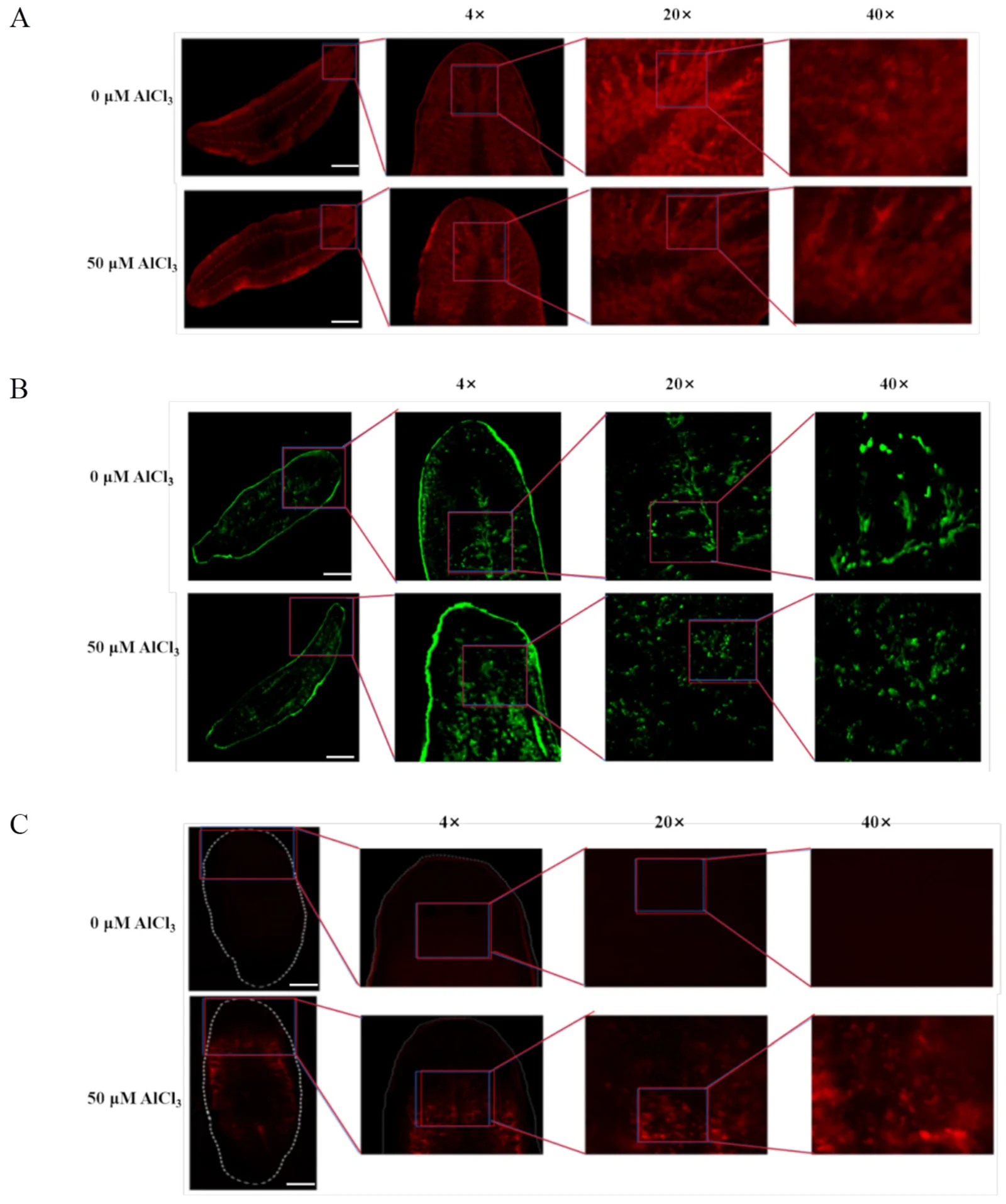
AlCl_3_ exposure impairs the neural structure of planarians. (**A**) Anti-SYNORF1 staining, (**B**) serotonergic (5-HT) neuron staining, and (**C**) apoptosis staining. Scale bars: 100 μm for (**A**,**B**), and 50 μm for (**C**).

The planarian CNS was visualized using anti-synapsin antibody (anti-SYNORF1) staining. The CNS of planarians consists of a pair of cerebral ganglia and two ventral nerve cords that extend through the entire body, and the nerve cords are interconnected via short nerve connections (Figure 6A). The fluorescence intensity and nerve fiber density of SYNORF1-immunopositive neurons in planarians exposed to 50 μM AlCl_3_ for 48 h were weaker and decreased compared with normal planarians (Figure 6A). This result demonstrates that 50 μM AlCl_3_ damaged the neural structure of planarians. We used a 5-HT-specific antibody to stain the planarians. After exposure to 50 μM AlCl_3_ for 48 h, the green fluorescence intensity in the planarians was significantly increased (Figure 6B). Finally, we used the one-step TUNEL apoptosis assay kit to stain the planarians and evaluate the effect of AlCl_3_ on planarian cell apoptosis (Figure 6C). We found that exposure to 50 μM AlCl_3_ for 48 h resulted in severe cell apoptosis in planarians compared with the normal group.

### 2.4. AlCl_3_ Induces an Imbalance of Neurotransmitters in Planarians

**Figure 7:**
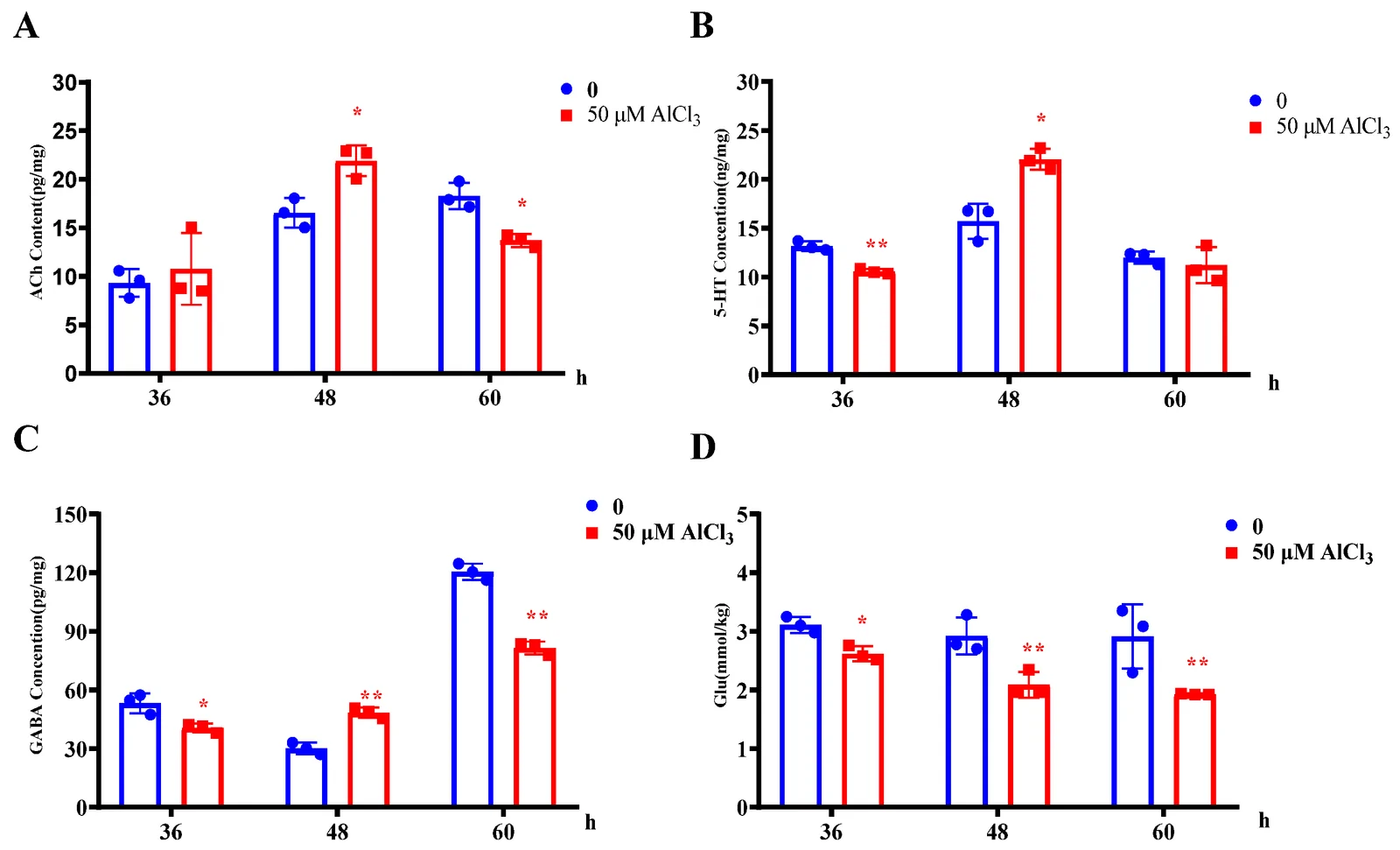
AlCl_3_ exposure induces an imbalance of neurotransmitters. (**A**) Acetylcholine (ACh), (**B**) 5-hydroxytryptamine (5-HT), (**C**) γ-aminobutyric acid (GABA), and (**D**) glutamate (Glu). Data are presented as the mean ± SD. N = 3, * *p* < 0.05 and ** *p* < 0.01 compared to the 0 μmol/L AlCl_3_ group; Student’s *t*-test, GraphPad 8.4.3.

Stable and balanced levels of neurotransmitters form the basis of normal neural activity. We measured the content of the neurotransmitters ACh, 5-HT, GABA, and Glu, which are closely related to cognitive function, to evaluate the influence of AlCl_3_ (Figure 7A–D). After exposure to AlCl_3_, the neurotransmitter content fluctuated significantly at 36, 48, and 60 h. For ACh, there was no difference at 36 h, a significant increase at 48 h and a significant decrease at 60 h (Figure 7A). For 5-HT, there was a significant decrease at 36 h, a significant increase at 48 h and no difference at 60 h (Figure 7B). For GABA, there was a significant decrease at 36 h and 60 h and a significant increase at 48 h (Figure 7C). The content of Glu decreased significantly at 36, 48, and 60 h (Figure 7D). These results reveal that AlCl_3_ induces neurotransmitter imbalances that impair cognitive function.

### 2.5. AlCl_3_ Causes Apoptosis in Planarians

**Figure 8:**
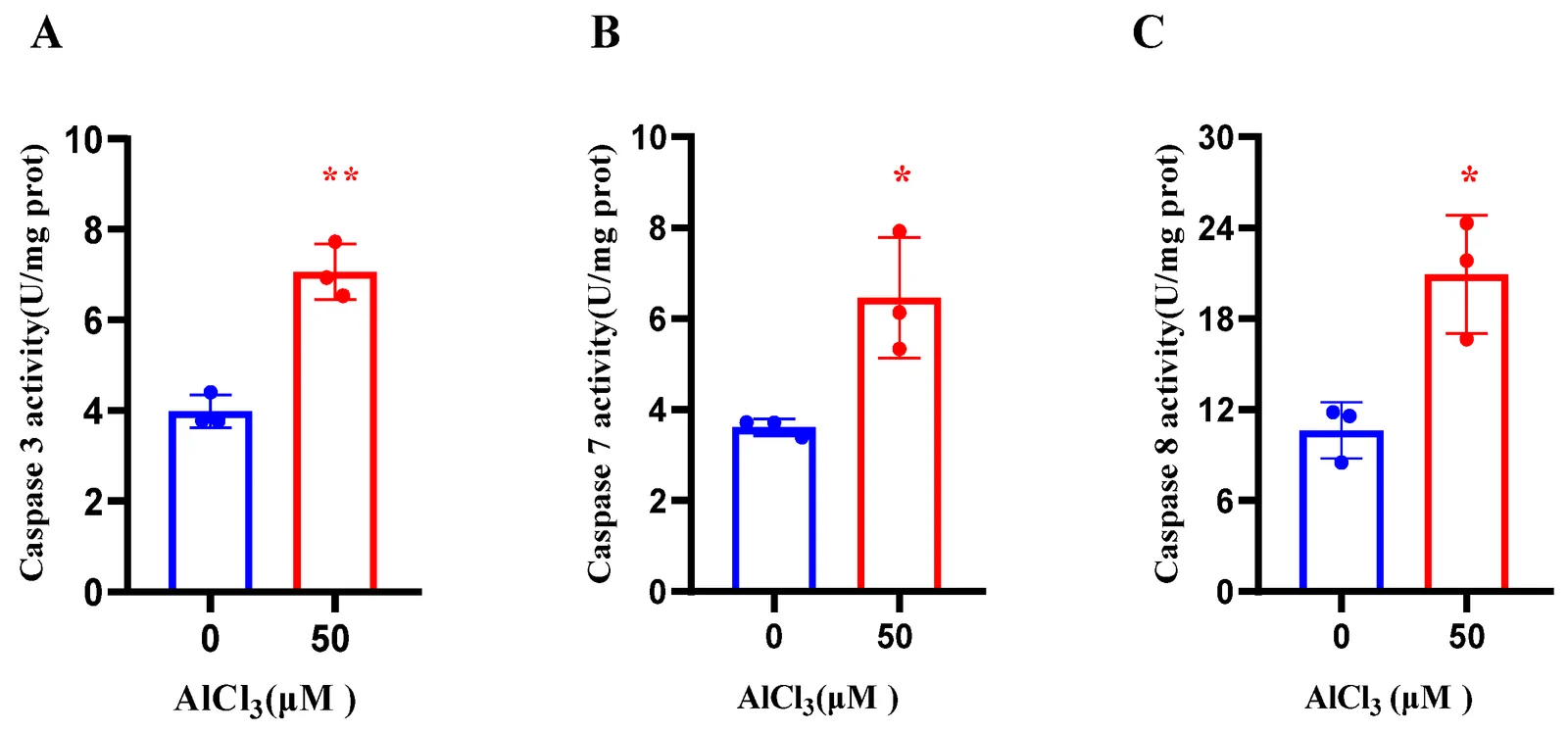
AlCl_3_ exposure promotes apoptosis in planarians. (**A**) Caspase-3, (**B**) caspase-7, and (**C**) caspase-8. Data are presented as the mean ± SD. N = 3, * *p* < 0.05 and ** *p* < 0.01 compared to the 0 μmol/L AlCl_3_ group; Student’s *t*-test, GraphPad 8.4.3.

We confirmed through TUNEL staining that AlCl_3_ can promote cell apoptosis in planarians (Figure 6C). We found that exposure to 50 μM AlCl_3_ for 48 h resulted in severe cell apoptosis in planarians compared with the normal group. At the same time, we also measured the activities of apoptosis-related enzymes. After exposure to AlCl_3_, the activities of caspase-3, caspase-7 and caspase-8 were significantly elevated (Figure 8A–C). The increase in apoptosis-related enzyme activity led to cell apoptosis, which in turn destroyed cells throughout the planarians, including nerve cells.

### 2.6. AlCl_3_ Induces Oxidative Stress in Planarians

**Figure 9:**
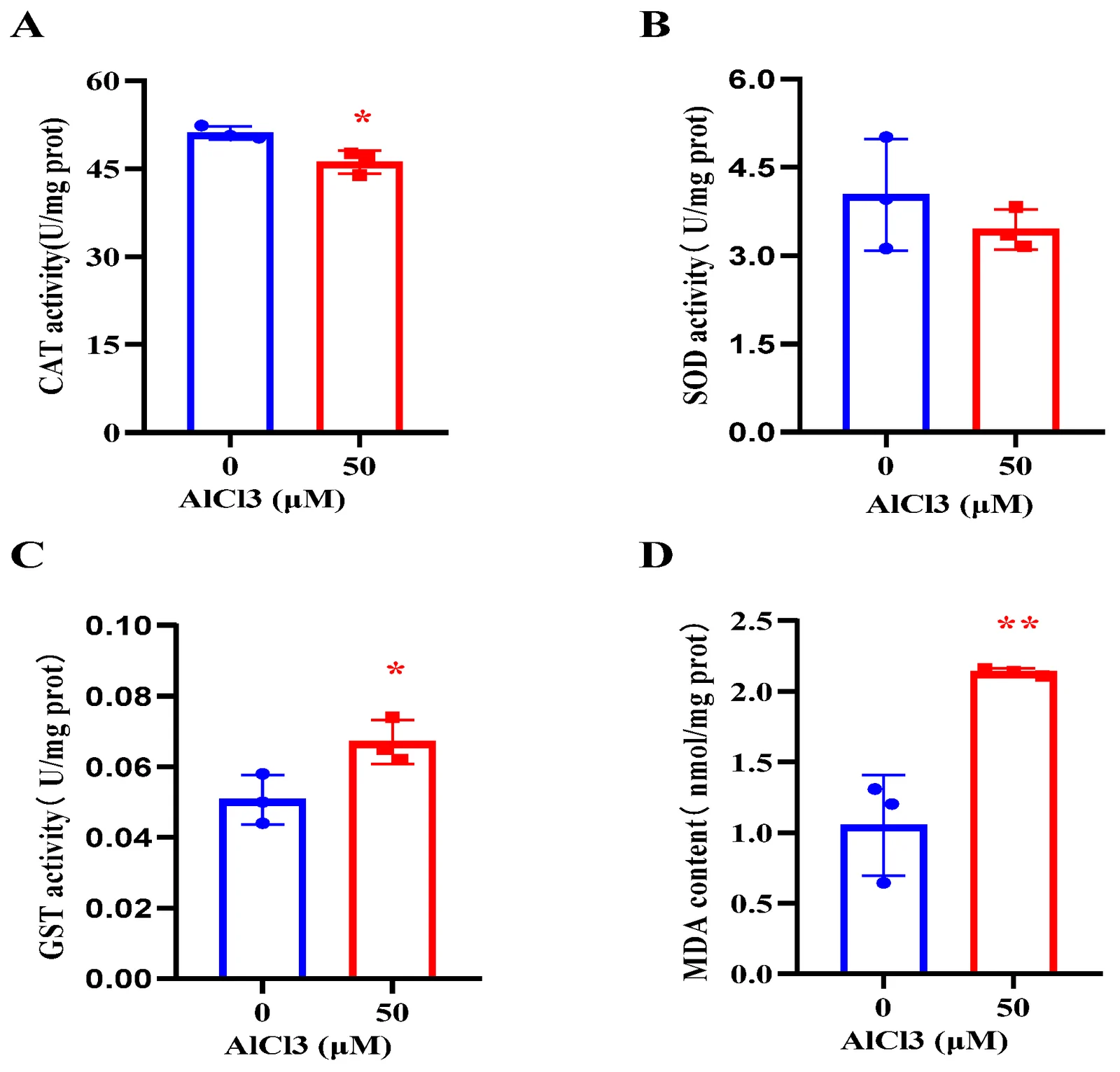
AlCl_3_ exposure promotes oxidative stress in planarians. (**A**) Catalase (CAT), (**B**) superoxide dismutase (SOD), (**C**) glutathione S-transferase (GST), and (**D**) malondialdehyde (MDA). Data are presented as the mean ± SD. N = 3, * *p* < 0.05 and ** *p* < 0.01 compared to the 0 μmol/L AlCl_3_ group; Student’s *t*-test, GraphPad 8.4.3.

We measured the activities of antioxidant enzymes in planarians to evaluate the effect of AlCl_3_ (Figure 9). After exposure to 50 μM AlCl_3_ for 48 h, the activity of CAT significantly declined (Figure 9A, *p* < 0.05). The activity of SOD showed no difference (Figure 9B). GST activity and MDA content were significantly increased (Figure 9C, *p* < 0.05; Figure 9D, *p* < 0.01). These results reveal that AlCl_3_ exacerbates oxidative stress in planarians, leading to cellular damage and lipid peroxidation.

### 2.7. Donepezil Alleviated the Cognitive Impairment Caused by AlCl_3_ in Planarians

**Figure 10:**
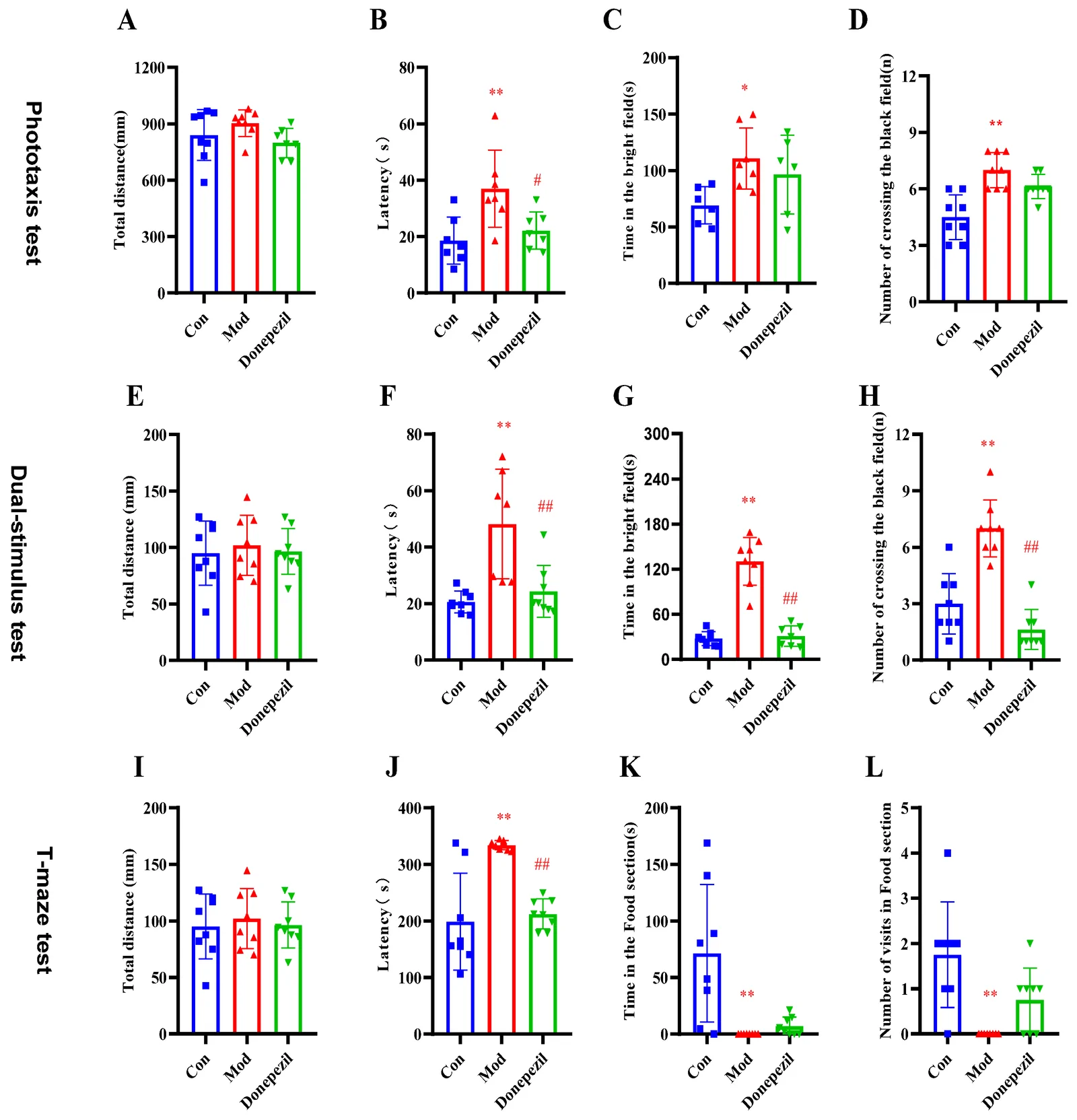
Donepezil alleviated the cognitive impairment caused by AlCl_3_ in planarians. Phototaxis test: (**A**–**D**). Dual-stimulus decision test: (**E**–**H**). T-maze test: (**I**–**L**). (**A**,**E**,**I**) Total distance. (**B**,**F**,**J**) Latency. (**C**,**G**,**K**) Time spent in the bright field. (**D**,**H**,**L**) Numbers. Data are presented as the mean ± SD. *n* = 8, * *p* < 0.05 and ** *p* < 0.01 compared to Con. # *p* < 0.05 and ## *p* < 0.01 compared to Mod. Student’s *t*-test, GraphPad 8.4.3.

Donepezil is a drug used clinically for the treatment of Alzheimer’s disease. We used donepezil to evaluate the cognitive dysfunction in a planarian model induced by AlCl_3_. We selected a dose of 7 μM donepezil as the optimal administration concentration. Using the behavioral testing method we established, we conducted the phototaxis test, dual-stimulus decision test, and T-maze test on planarians after administration of donepezil (Figure 10). In any of the behavioral tests, there was no difference in the total distance traveled by the planarians (Figure 10A,E,I). Among the three behavioral tests, the latency of the AlCl_3_ group (Mod) was significantly prolonged, while that of the donepezil treatment group was significantly shortened (Figure 10B,F,J, *p* < 0.01). In the phototaxis test, the time spent in the bright field and the number of crossings into the dark field in the Mod group were significantly increased (Figure 10C, *p* < 0.05; Figure 10D, *p* < 0.01). After treatment with donepezil, the time spent in the bright field and the number of crossings into the dark field showed a downward trend (Figure 10C,D). In the dual-stimulus decision test, donepezil significantly reduced the time spent in the bright field and the number of crossings into the dark field (Figure 10G, *p* < 0.01; Figure 10H, *p* < 0.01). At the same time, in the T-maze test, donepezil reversed the downward trend in the time spent in the food section and the number of visits by the planarians (Figure 10K,L). These results indicated that donepezil alleviated the cognitive impairment caused by AlCl_3_ in planarians.

## 3. Discussion

In this study, we established a new model of cognitive dysfunction in planarians induced by AlCl_3_. We conducted behavioral studies using a phototaxis test, dual-stimulus decision test and T-maze test, and the results confirmed that the cognitive function of planarians was impaired. Immunohistochemical and immunofluorescence experiments confirmed that AlCl_3_ damaged the neural structure of planarians. AlCl_3_ disrupted the balance of neurotransmitters, leading to cell apoptosis and oxidative stress in planarians. Finally, we confirmed that donepezil alleviated the cognitive impairment caused by AlCl_3_ in planarians.

Planarians are very sensitive to the external environment, and research has been conducted on their application in the field of environmental toxicity [25,26]. In order to find an AlCl_3_ concentration that did not harm the survival status of planarians but did impair cognitive function, we set the AlCl_3_ concentrations at 10, 25, 50, 100, and 200 μM. To quantify the survival status of the planarians, we developed a status score for them based on previous studies [37,38]. The scoring results indicate that 200 μM AlCl_3_ caused severe damage. This was consistent with the conclusion reported in the literature that the toxicity to planarians increases as the concentration of toxic substances rises.

It has been reported that planarians have cognitive functions [27,28,29]. We conducted three behavioral experiments: the phototaxis test, dual-stimulus decision test and T-maze test. By integrating the results of these three behavioral tests, we found that AlCl_3_ leads to cognitive impairment (Figure 2, Figure 3 and Figure 4). Behavioral studies in planarians have been reported [28,29]. The toxicity of chemical substances can be evaluated by observing the state of planarians. By using the method of counting squares, the locomotor ability of the planarians was studied [39]. A digital video camera and planarian behavior analysis software were used for binary competitive behavior analyses [40]. Similarly, we utilized the XR-Fish2 software for planarian ethology to record the movement trajectories of the planarians. The rapid collection and analysis of experimental data have significantly enhanced the efficiency of the experiments. Previous studies only focused on the status and individual behavior of planarians, without conducting a comprehensive analysis of multiple behaviors. In addition to the phototaxis test and dual-stimulus decision test, we designed a T-maze experiment for planarians based on previous literature in order to further investigate the learning and memory abilities of planarians [29]. Planarians have similar locomotor characteristics to those of mammals, making it possible for us to attempt to study the cognitive functions of planarians through behavioral research.

Through immunohistochemical and immunofluorescence experiments, we found that after exposure to AlCl_3_, the nervous system of planarians was impaired. HE staining showed inflammation in the planarians. Nissl staining indicated a decrease in neuronal vitality. The planarian CNS was visualized using anti-synapsin antibody (anti-SYNORF1) staining. The CNS of planarians consists of a pair of cerebral ganglia and two ventral nerve cords that extend through the entire body, and the nerve cords are interconnected via short nerve connections. This is consistent with reports in the literature [25,41]. This result demonstrates that 50 μM AlCl_3_ damaged the neural structure of planarians. Planarians have 5-HT neurons that regulate neural activity and are closely related to their cognitive functions [39]. 5-HT neuron staining showed that exposure to AlCl_3_ significantly increased 5-HT neuron density. AlCl_3_ caused damage to the neural structure, induced inflammation and reduced the vitality of neurons. Lastly, we found via TUNEL staining that exposure to 50 μM AlCl_3_ for 48 h resulted in severe cell apoptosis in planarians. Such phenomena have also been observed in other animals such as mice and zebrafish [34,35]. The same has also been observed in planarians. This indicates that planarians provide a material basis for the study of cognitive function models.

Stable and balanced levels of neurotransmitters form the basis of normal neural activity. We measured the contents of the neurotransmitters ACh, 5-HT, GABA, and Glu, which are closely related to cognitive function. This was consistent with reports that toxic substances can cause changes in individual neurotransmitters, such as ACh [42] and 5-HT [39]. However, the reports only focused on changes in the content of a single neurotransmitter and did not conduct an analysis of the fluctuations of multiple neurotransmitters over time. This was determined by the purpose of their research. Measuring the content of neurotransmitters using whole-body homogenates of planarians has certain limitations. Whole-body homogenates cannot provide neuron-specific concentrations, and future studies using neuron-enriched fractions or neuron-specific markers would be valuable to further validate these findings.

After exposure to AlCl_3_, the activities of caspase-3, caspase-7 and caspase-8 were significantly elevated (Figure 7A–C). This was consistent with our TUNEL staining results. The increase in apoptosis-related enzyme activity led to cell apoptosis, which in turn destroyed cells throughout the planarians, including nerve cells. It should be noted that the TUNEL assay and caspase activity measurements used in this study detect general apoptosis in whole-body homogenates or tissue sections and cannot distinguish neuronal apoptosis from apoptosis of other cell types. Therefore, the observed increase in apoptosis may involve neuronal cells, but is not necessarily neuron-specific. Co-localization of TUNEL or activated caspase-3 with neuronal markers, together with quantification of the proportion of affected neurons, will be necessary to confirm and quantify neuronal apoptosis in future studies.

At the same time, the activities of CAT, SOD, and GST and the content of MDA were measured. The results revealed that AlCl_3_ exacerbated oxidative stress in planarians, leading to cellular damage and lipid peroxidation.

Donepezil is a drug used clinically for the treatment of AD. We used donepezil to evaluate cognitive dysfunction induced by AlCl_3_ in a planarian model. The results of the phototaxis test, dual-stimulus decision test and T-maze test indicated that donepezil alleviated the cognitive impairment caused by AlCl_3_ in planarians. This indicates that the model we established for investigating cognitive dysfunction in planarians was feasible.

This model only reproduces some pathological and behavioral features associated with AD, rather than fully reproducing AD pathology. The planarian model has several inherent limitations. First, planarians are invertebrates and lack the Aβ plaques and tau neurofibrillary tangles that are hallmark pathological features of AD. Second, their nervous system is considerably simpler than that of mammals, and they do not possess a true blood–brain barrier or mammalian-like pharmacokinetic and metabolic profiles. Finally, although planarians display phototaxis, decision-making and T-maze learning, these behaviors represent only a limited repertoire of cognitive-like functions and cannot fully capture the complex cognitive domains affected in AD. Therefore, the planarian model should be considered a complementary high-throughput screening tool for identifying compounds that may alleviate cognitive impairment. The findings obtained in this model require further validation in mammalian models and clinical studies.

## 4. Materials and Methods

AlCl_3_ was purchased from Shanghai Maclean Biochemical Technology Co., Ltd. (Shanghai, China). Catalase (CAT) activity assay kit, superoxide dismutase (SOD) activity assay kit, glutathione S-transferase (GST) activity assay kit, malondialdehyde (MDA) content assay kit, hematoxylin-eosin (HE) staining kit and Nissl staining kit were purchased from Beijing Solab Technology Co., Ltd. (Beijing, China). Neurotransmitter assay kits for the detection of acetylcholine (ACh), γ-aminobutyric acid (GABA), serotonin (5-HT) and glutamate (Glu) were purchased from Reed Biotech Co., Ltd. (Wuhan, China). Caspase activity assay kits for caspase-3, caspase-7 and caspase-8 were purchased from Elabscience Biotechnology Co., Ltd. (Wuhan, China). The One-step TUNEL apoptosis assay kit was obtained from Beyotime Biotechnology (Shanghai, China). The anti-SYNORF1 antibody was obtained from Developmental Studies Hybridoma Bank (Iowa City, IA, USA). The anti-SERT antibody was obtained from Wuhan Sanying Biotechnology (Wuhan, China). All other chemicals and reagents were of the highest analytical grade and were commercially available. Beef liver was purchased from a local slaughterhouse.

### 4.1. Planarian Maintenance

The planarians (Dugesia ZB) utilised were provided by Professor Zhenbiao Xu of the Shandong University of Technology [43]. We then carried out planarian asexual reproduction. The planarians were raised in incubators held at a constant temperature of 22 °C and a humidity of 70~80% in dechlorinated water that had been exposed to the air for 24 h. The food of the planarians was crushed fresh beef liver, given once a week. The water was changed immediately after the planarian feeding was completed. The water was changed every two days at other times. Planarians 8–10 mm in length were selected and starved for 7 days before the experiments in order to ensure that they were maintained in a balanced metabolic state. All planarians were maintained and handled according to a protocol approved by the Animal Care and Use Committee of Shandong University of Traditional Chinese Medicine. Every effort was made to minimize the number of animals used and their suffering. We confirm that the authors complied with the ARRIVE guidelines.

### 4.2. Exposure to AlCl_3_

In the experiment, planarians from the same batch that had propagated asexually and measured 8–10 mm in length were selected. The planarians were randomly assigned to the control group or AlCl_3_ groups. Each group consisted of 5–8 individuals (*n* = 5 or *n* = 8), and three parallel groups (N = 3) were set up for each group. For the planarian survival test, the scores of three parallel groups (N = 3, *n* = 5) with 15 planarians were recorded and calculated. One group was randomly selected from the three parallel groups for behavioral testing (*n* = 8). For biochemical analyses, N = 3 indicates the number of independent biological replicates per group. Each biological replicate consisted of a pooled sample of 8 planarians from one independent culture plate. The samples were homogenized separately, and each homogenate was measured as one biological replicate. For histopathological sectioning, one planarian was randomly selected from each concentration group.

Planarians were transferred to 6-well plates. AlCl_3_ solutions were prepared at concentrations of 10, 25, 50, 100, and 200 μM using ultrapure water. After being starved for 7 days, the control group was transferred to water, and other groups were transferred to different concentrations of AlCl_3_. The culture medium was replaced at 24 h intervals. We evaluated the condition of the planarians according to the standards to study the effect of AlCl_3_ on planarians.

### 4.3. Phototaxis Test

Planarians tend to avoid strong light and prefer weak light. The response of planarians to light can reflect the physiological state of the organism [28,40]. We conducted a phototaxis test to study the effect of AlCl_3_ on planarians. We designed a black box with dimensions of 40 cm in length, 20 cm in width and 30 cm in height. A Petri dish 10 cm in diameter was positioned at the bottom center of the box. The box was divided into a light chamber (L) and a dark chamber (D) by a black plastic plate. There was a gap in the center of the black plastic plate, which ensured that the planarians could pass through freely. The light intensity of the light chamber (L) was set at 1000 lx. The planarians to be tested were placed in the light chamber area of the Petri dish and allowed to move freely for 3 min; then the gap was opened. The duration of the test was six minutes. XR-Fish2 software for planarian ethology was used to record and analyse the movement trajectories of the planarians. The total distance, the latency time to enter the dark chamber (D), duration of stay in the light chamber, and number of crossings into the dark chamber were analysed to assess the impact of AlCl_3_ on the phototaxis of planarians.

### 4.4. Dual-Stimulus Decision Test

Inoue’s work indicates that planarians show decision-making behavior in response to multiple stimuli through integrative brain function. When the brain is damaged, this function declines [28]. Therefore, we also used the dual-stimulus decision test to evaluate the effect of AlCl_3_ on planarians. We used the same test setup as in the phototaxis test. The difference was that the crushed fresh beef liver for the planarians was placed in the center of the light chamber (L). The planarians to be tested were placed in the light chamber of the Petri dish and allowed to move freely for a period of three minutes. The food was then placed in the center of the light chamber (L). The duration of the test was six minutes. The XR-Fish2 software for planarian ethology was used to record the movement trajectories of the planarians. The total distance, the latency time to enter the dark chamber (D), duration of stay in the light chamber, and number of crossings into the dark chamber were analysed to assess the impact of AlCl_3_ on dual-stimulus decision functions of planarians.

### 4.5. T-Maze Test

Maze-type experiments are often used to test the learning and memory functions of animals. We designed the T-maze experiment for planarians based on the T-maze experiment of zebrafish [44,45]. The T-maze apparatus was designed in the shape of the English letter T. The dimensions of the maze were as follows: 15 centimetres in length, 3 centimetres in width, and 2 centimetres in height. A food reward chamber (FC zone) containing bovine liver was placed at the end of the left horizontal arm. Each group of planarians was subjected to three training trials, with an interval of one hour between each. Planarians were acclimated to the T-maze environment for three minutes before the test began. During the training period, food was placed in the FC zone. If a planarian was unable to enter the FC zone, we provided guidance. During the testing period, the food in the FC zone was removed. The XR-Fish2 software for planarian ethology was used to record the movement trajectories of the planarians. The total distance, the latency time to first enter the FC zone and the duration of time in the FC zone were analysed to assess the impact of AlCl3 on the learning and memory functions of planarians.

### 4.6. Immunohistochemistry

The planarians were treated with 2% hydrochloric acid, followed by fixation, washing, dehydration, rehydration, wax impregnation, embedding, and sectioning to prepare the paraffin sections. The thickness of the resulting sections was 5 μm. HE staining and Nissl staining were performed according to the manufacturers’ instructions using HE staining and Nissl staining kits. 5-HT neuron staining was carried out using anti-SERT. The planarian sections were dewaxed with xylene and ethanol, washed with distilled water, and incubated in a citrate buffer solution for antigen retrieval. Sections were then incubated with 5% goat serum at 37 °C for 0.5 h and then with anti-SERT (1:400) overnight at 4 °C. The sections were then incubated with goat anti-rabbit 488 (1:1500) at room temperature for 1 h. All stained sections were observed under a microscope (Keyence Microscope, BZ-X800E, KEYENCE CORPORATION, Osaka, Japan).

### 4.7. Immunofluorescence

The planarians were treated with 2% hydrochloric acid. The samples were then fixed with 4% paraformaldehyde fixing solution for 4 h. The staining was performed using a TUNEL staining kit according to the manufacturer’s instructions. The samples were rinsed three times with PBS and then incubated with 0.3% Triton X-100 in PBS for 5 min. TUNEL reagent was then added, and the samples were incubated at 37 °C in the dark for 1 h. The paraformaldehyde-fixed planarians were washed three times with PBS and then blocked with 10% skimmed milk for 2 h. Anti-SYNORF1 and dylight 594 antibodies were added in sequence. After washing with PBS, the samples were sealed with glycerol and observed with an inverted fluorescence microscope.

### 4.8. Neurotransmitter Analysis

In order to assess the influence of AlCl_3_ on the neurons of planarians, we measured the Glu, ACh, 5-HT and GABA contents using ELISA assay kits after the planarians were treated with AlCl_3_. The whole planarians were used. Each biological replicate consisted of a pooled sample of 8 planarians from one independent culture plate. The samples were homogenized in PBS, and the homogenate was centrifuged at 12,000 rpm for 10 min at 4 °C to obtain the supernatant. Before measuring neurotransmitter and other levels, the total protein concentration of each supernatant was determined using a BCA protein assay kit according to the manufacturer’s instructions. The total protein concentrations of the samples were normalized. Then, the content of neurotransmitters was measured using ELISA assay kits according to the manufacturer’s instructions. The supernatants were then used to measure the neurotransmitter levels at 450 nm using a Multiskan FC Microplate Reader (Thermo Fisher, Waltham, MA, USA) according to the manufacturer’s protocol.

### 4.9. Apoptosis Analysis

We evaluated the effect of AlCl_3_ on apoptosis by detecting the activity of apoptosis-related enzymes. We measured the activity of caspase-3, caspase-7 and caspase-8 using a commercial test kit. The activity was measured using an ELISA assay kit according to the manufacturer’s instructions. The supernatants of planarian homogenates were used to measure enzyme activity using a Multiskan FC Microplate Reader (Thermo Fisher, Waltham, MA, USA) according to the manufacturer’s protocol.

### 4.10. Oxidative Stress Analysis

To assess oxidative stress in planarians after exposure to AlCl_3_, the activities of CAT, SOD, and GST, as well as MDA content, were measured using assay kits, according to the manufacturer’s instructions. The supernatants of planarian homogenates were used to measure enzyme activity using a Multiskan FC Microplate Reader (Thermo Fisher, Waltham, MA, USA).

### 4.11. Donepezil Alleviated the Cognitive Impairment Caused by AlCl_3_ in Planarians

Planarians 8–10 mm in length were selected and starved for 7 days before the experiments. The planarians were divided into the control group, the model group and the donepezil group. Each group consisted of 8 individuals, and three parallel groups were set up. The planarians of the control group were in dechlorinated water. The planarians in the model group were exposed to 50 μM AlCl_3_. The planarians in the donepezil group were exposed to 50 μM AlCl_3_ for 12 h, and donepezil was then added to the solution for 36 h. Finally, we evaluated the behavior of the planarians to assess the therapeutic effect of donepezil.

### 4.12. Statistical Analysis

All data are expressed as mean ± SD. The criterion for excluding data was that data more than 2 standard deviations were considered outliers. For statistical analysis, differences between two groups were assessed using Student’s *t*-test. Comparisons among multiple groups were made using one-way ANOVA followed by Dunnett’s multiple comparisons test. The statistical analyses were performed using GraphPad Prism software version 8.4.3, and *p* < 0.05 was established as the threshold for statistical significance.

## 5. Conclusions

In summary, a new model of cognitive dysfunction in planarians induced by AlCl_3_ was established. AlCl_3_ impaired the cognitive function of planarians, damaged their neural structure, disrupted the balance of neurotransmitters, and aggravated cell apoptosis and oxidative stress. However, this model only reproduces some pathological phenotypes of Alzheimer’s disease. Therefore, the planarian model can be considered a promising complementary platform for high-throughput screening of candidate drugs for cognitive impairment.

## Figures and Tables

**Table 1:** The scoring criteria for the survival status of planarians.

Evaluation Metrics	State 1	State 2	State 3	State 4	State 5
Planarian	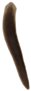	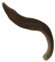	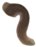	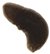	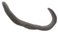
Status	stretches	twist	S-like shaped	shrinks	C-like shaped
Score	10–9	8–7	6–5	4–3	2–0

## Data Availability

The data presented in this study are available within the article and Supplementary Materials.

## Supplementary Materials

The following supporting information can be downloaded at: https://www.mdpi.com/article/10.3390/ijms27209364/s1.
