# Supplementary material for: Aluminum Chloride-Induced Planarian Model with Cognitive Impairments Through Neuronal Damage, Neurotransmitter Imbalance, Apoptosis, and Oxidative Stress
Source: Int J Mol Sci. 2026 Oct 21;27(20):9364. doi: 10.3390/ijms27209364 (PMC13704994; doi:10.3390/ijms27209364)
Supplement: Supplementary file 1 [file ijms-27-09364-s001.zip › ijms-4597545-supplementary.pdf]

Supplemental Information

Aluminum chloride induced planarian model with cognitive impairments through neuronal damage, neurotransmitters imbalance, apoptosis, oxidative stress

Qingxian Wang[1,†], Shixue Wang[1,†], Zhenbiao Xu[2], Linxia Song[2], Kexin Yang[2], Chunmei Zhang[2], Xiaorui Cheng[1,*]

[1] Innovative Institute of Chinese Medicine and Pharmacy, Shandong University of Traditional Chinese Medicine, Jinan 250355, China

[2] School of Life and Medicine, Shandong University of Technology, Zibo, 255000, China

†These authors have contributed equally to this work and share first authorship.

*Correspondence: Xiaorui Cheng, 60011982@sdutcm.edu.cn.

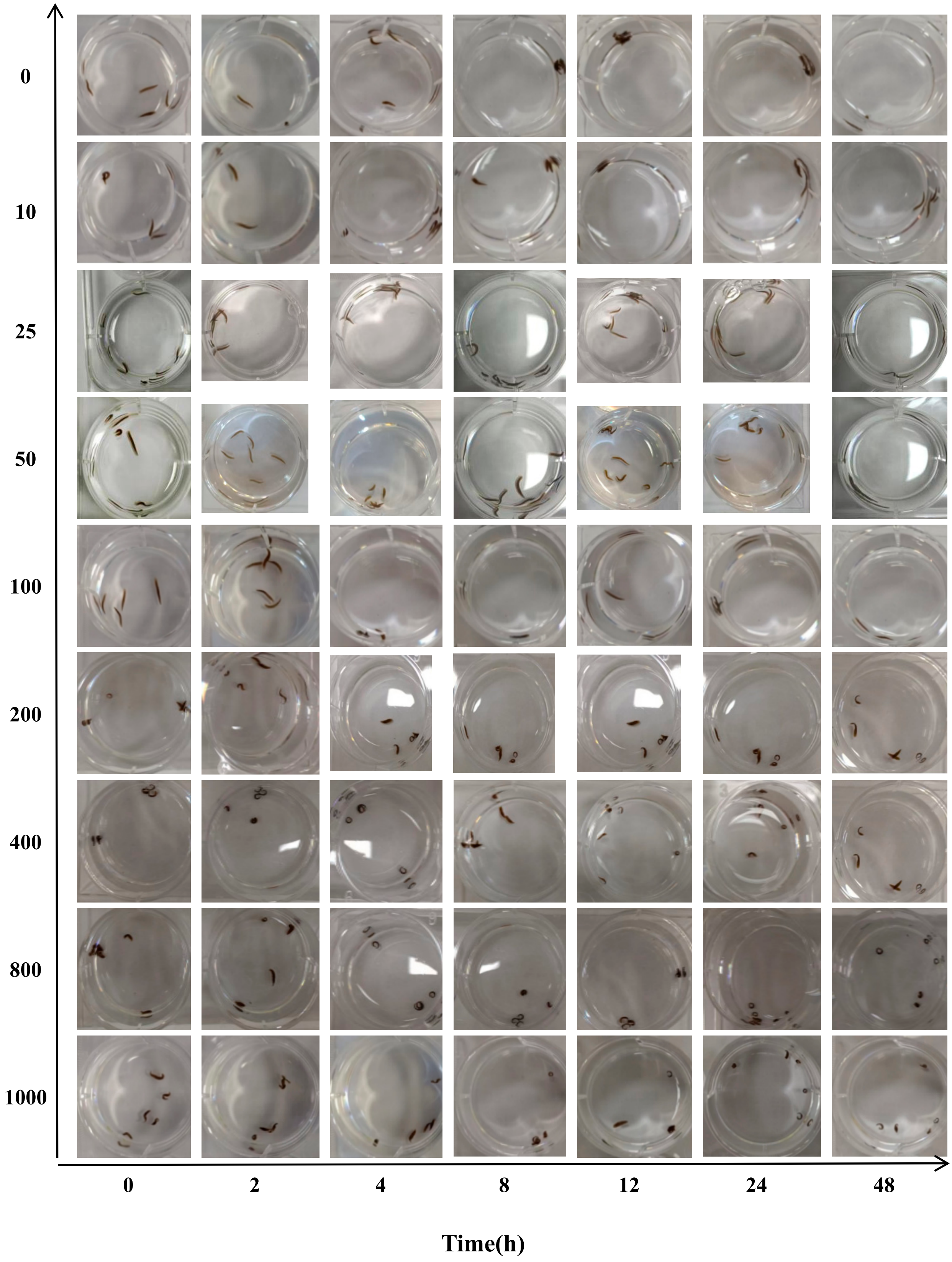

Figure S1 Digital photos of planarian in different concentration of $AlCl_3$ at different time

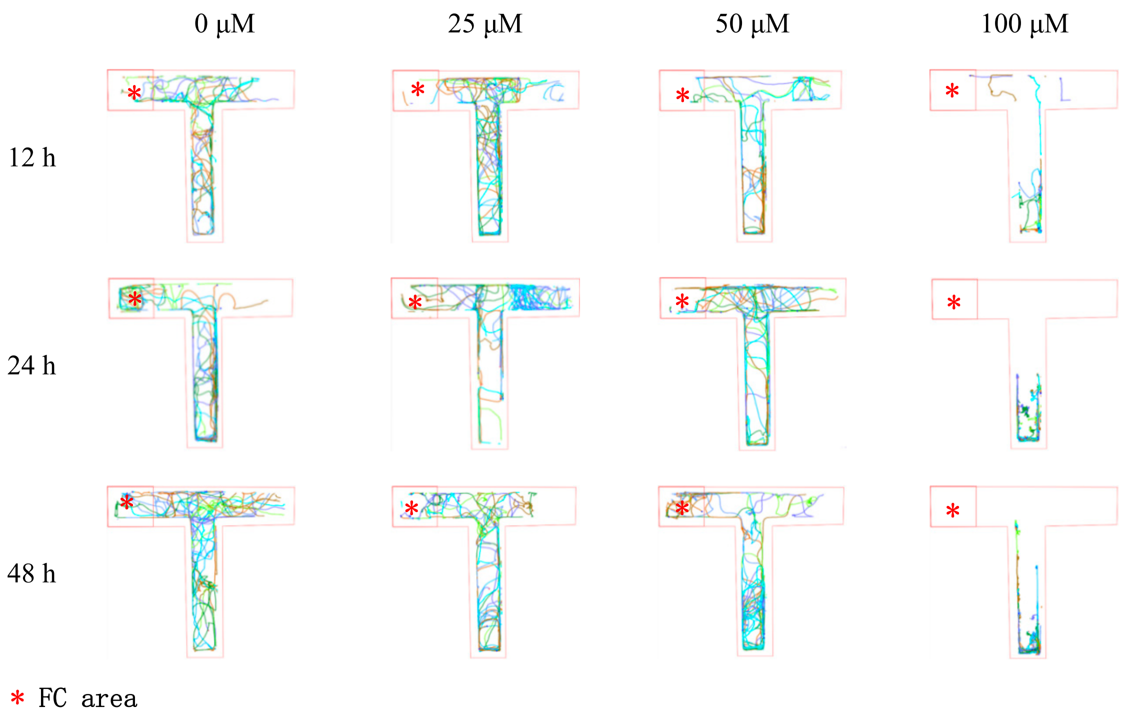

Figure S2 The movement trajectory diagram of the planarian of T-maze test

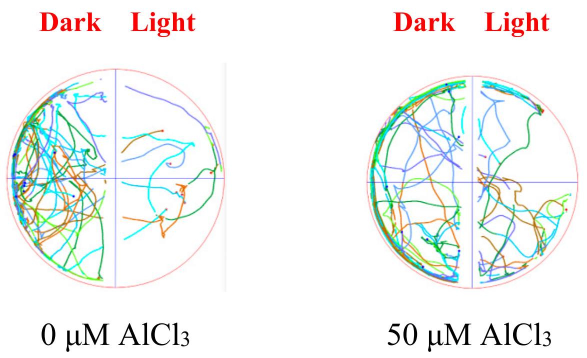

Figure S3 The movement trajectory diagram of the planarian of phototaxis test

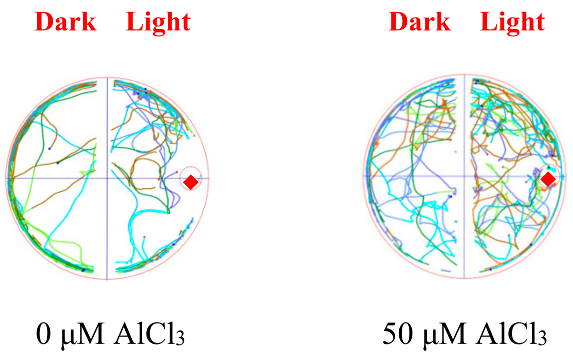

◆ The food location

Figure S4 The movement trajectory diagram of the planarian of dual-stimulus decision test

<table>
<tr><th>No data exclusion</th><th>Data exclusion based on a 2 SD criterion</th></tr>
<tr><td>Figure 2A</td><td>Figure 2A</td></tr>
<tr><td>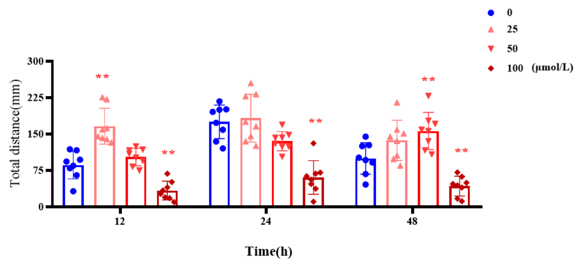</td><td>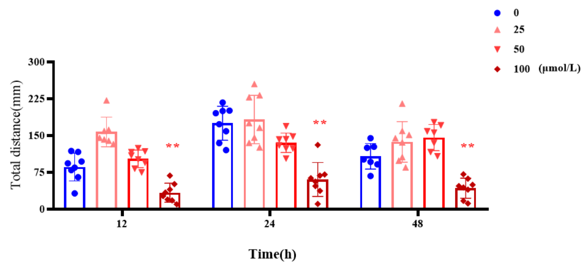</td></tr>
<tr><td>Figure 3A</td><td>Figure 3A</td></tr>
<tr><td>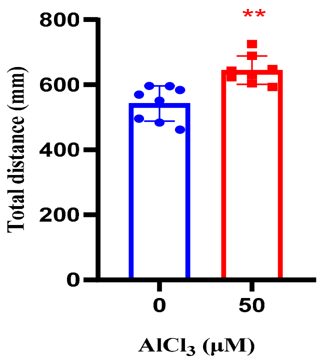</td><td>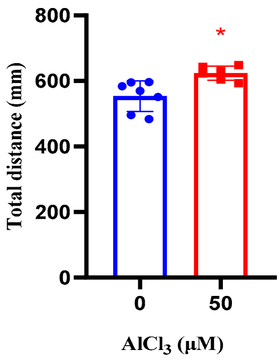</td></tr>
<tr><td>Figure 4A</td><td>Figure 4A</td></tr>
<tr><td>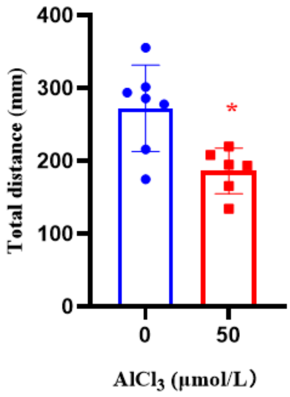</td><td>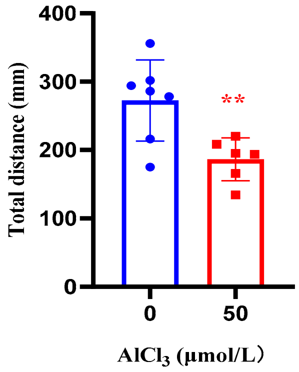</td></tr>
</table>

Figure S5 Comparison of statistical results before and after excluding data by two standard deviations
